# Klinefelter Syndrome: A Genetic Disorder Leading to Neuroendocrine Modifications and Psychopathological Vulnerabilities in Children—A Literature Review and Case Report

**DOI:** 10.3390/children11050509

**Published:** 2024-04-24

**Authors:** Fabiola Panvino, Roberto Paparella, Luisiana Gambuti, Andrea Cerrito, Michela Menghi, Ginevra Micangeli, Carla Petrella, Marco Fiore, Luigi Tarani, Ignazio Ardizzone

**Affiliations:** 1 Department of Human Neuroscience, Section of Child and Adolescent Neuropsychiatry, Sapienza University of Rome, Via dei Sabelli 108, 00185 Rome, Italy; 2 Department of Maternal Infantile and Urological Sciences, Sapienza University of Rome, Viale del Policlinico 155, 00161 Rome, Italy; 3 Institute of Biochemistry and Cell Biology, National Research Council (IBBC-CNR), 00185 Rome, Italy

**Keywords:** Klinefelter syndrome, psychopathology, prepubertal, depression, anxiety, mosaicism

## Abstract

Klinefelter syndrome (KS), characterized by an additional X-chromosome in males, manifests in a wide range of neuroendocrine and psychiatric symptoms. Individuals with KS often face increased risks of hormonal dysfunction, leading to depression and anxiety, although extended research during pediatric and adolescent age is still limited. This critical phase, decisive for KS children, is influenced by a combination of genetic, environmental and familial factors, which impact brain plasticity. In this report, we reviewed, in a narrative form, the crucial KS psychopathological hallmarks in children. To better describe neuroendocrine and neuropsychiatric outcomes in children with KS, we presented the case of an 11-year-old prepubertal child with mosaic KS who was referred to our Center of Developmental Psychopathology due to a decline in his academic performance, excessive daytime fatigue and increased distractibility over the past few months. Family history revealed psychiatric conditions among first- and second-degree relatives, including recently divorced parents and a 15-year-old sister. Early-onset persistent depressive disorder and anxious traits were diagnosed. Timely identification of susceptible children, with thorough examination of familial psychiatric history, environmental influences and neurocognitive profile, alongside targeted interventions, could potentially mitigate lifelong psychopathology-related disabilities in pediatric and adolescent KS cases, including those with mosaic KS.

## 1. Introduction

Klinefelter syndrome (KS) is the most common sex chromosomal anomaly in males, characterized by one or more extra X-chromosomes (typically 47,XXY), leading to testicular dysfunction and potential low fertility [1]. While the classic symptoms in adults include small testes, gynecomastia and infertility, KS presents a diverse range of clinical manifestations, which are often not evident until after puberty due to their non-specific nature in prepubertal stages [2,3]. Factors such as androgen receptor gene repeat, X-chromosome inactivation patterns and mosaicism contribute to this variability [4], often resulting in delayed or missed diagnoses until adulthood, primarily during infertility evaluation [5,6,7].

KS has been increasingly associated with neuroendocrine abnormalities and psychopathological vulnerabilities in affected children. Understanding the intricate interplay between genetic, neuroendocrine and psychological factors is fundamental for comprehensively addressing the challenges faced by these individuals. Early and accurate diagnosis is crucial for managing and preventing the various comorbidities associated with KS, including cardiovascular, metabolic and skeletal issues, which contribute to higher morbidity and mortality rates compared to the general male population [6,8,9].

The neuroendocrine system plays a pivotal role in regulating various physiological processes, including mood, cognition and behavior [10]. Subjects with KS often exhibit disruptions in this delicate hormonal balance, primarily attributed to hypogonadism and alterations in gonadal hormone levels. Testosterone deficiency, a hallmark feature of KS, can significantly impact brain development and function, predisposing individuals to a spectrum of neurobehavioral difficulties [11]. Additionally, KS patients are at greater risk of psychiatric conditions, such as anxiety, depression, schizophrenia and autism; there is also an association with attention deficit hyperactivity disorder (ADHD), which is notably more recognized and researched [1]. KS is not typically associated with global intellectual disability, distinguishing it from other non-sexual chromosome trisomies [12,13]. Studies on the neurocognitive and psychological profile in KS primarily focus on adult subjects, but the recent European Academy of Andrology guidelines on KS emphasize the importance of monitoring speech development, learning abilities and psychosocial issues in KS children and adolescents [1]. The enduring effects of neuroendocrine dysregulation and psychiatric comorbidities on educational attainment, employment prospects and interpersonal relationships among males with KS have been studied. Furthermore, the intersectionality of KS with other neurodevelopmental and psychiatric disorders, such as autism spectrum disorder and ADHD, underscores the complexity of the clinical presentation and management strategies required [14,15].

This article highlights, in a narrative form, the crucial KS psychopathological hallmarks in pediatric and adolescent age. Subjects with KS face unique challenges in terms of endocrine dysregulation having an impact on neurodevelopment and behavior. Specifically, hormonal imbalances associated with KS, such as testosterone deficiency, and the presence of an extra X-chromosome may influence brain development and contribute to neuropsychiatric vulnerabilities in KS populations. To better describe KS outcomes throughout childhood, we described the case of an 11-year-old boy with mosaic KS to illustrate how impaired school performance can have a multifactorial nature, serving as an early warning sign for potential psychopathological symptoms, which require prompt evaluation, even during the prepubertal period.

Moreover, targeted interventions aimed at addressing specific neurocognitive and psychosocial needs during childhood and adolescence may have far-reaching benefits in terms of long-term outcomes and adaptive functioning in adulthood. Therefore, while the focus of this narrative review centers on the critical psychopathological hallmarks of KS in pediatric and adolescent populations, it is essential to recognize the broader implications for lifelong neurodevelopmental trajectories and psychosocial well-being. By adopting a comprehensive and multidisciplinary approach, healthcare professionals can better support individuals with KS across the lifespan, ultimately promoting optimal health outcomes and quality of life. The literature for this narrative review was sourced from online databases, including Medline, Medline Complete, PubMed and Google Scholar, utilizing search terms such as Klinefelter syndrome, psychopathology, prepubertal, depression, anxiety, mosaicism.

## 2. Klinefelter Syndrome Psychopathological Hallmarks

### 2.1. Intelligence Quotient (IQ)

Individuals with KS typically achieve normal full-scale intelligence quotient (FSIQ) scores, averaging around 93 [16], though significantly lower than healthy controls [17]. An imbalance between performance IQ (PIQ) and verbal IQ (VIQ) has been reported as typical presentation of 47,XXY KS [18], possibly due to language-related deficits, which emerge early and intensify with academic demands [19]. In fact, as highlighted by previous research, both receptive and expressive language skills tend to be compromised [20], often exhibiting phonological deficits with greater semantic than phonemic fluency [21]. Conversely, a novel finding identified an alternative profile of 47,XXY KS with increased verbal capabilities in comparison with perceptual skills, as well as commensurate expressive and receptive vocabulary [22].

Parental education, prenatal/postnatal diagnosis and testosterone treatment collectively explain a significant portion of the variance in VIQ (43%) and PIQ scores (45%) among 47,XXY individuals, with higher parental education, prenatal diagnosis and testosterone treatment associated with better outcomes [23].

Recently, VIQ was found to be a predictor of mental health for KS men but not healthy controls [17], and an association between neuropsychological functions and sleep for 47,XXY men was established [18].

### 2.2. Specific Learning Disorders

Childhood academic struggles may signal specific learning disorders in KS or indicate emotional issues, such as anxiety and depression [24,25]. Up to 75% of KS individuals may have reading disorders [26], whereas mathematical deficits are less common [27]. Familial risk for specific learning disorders impacts KS cognitive phenotypes [28].

### 2.3. Executive Functions

Deficits in executive functions, social cognition and language are prominent features of neurocognitive profiles among males with KS [7,29,30]. Executive skills develop gradually, with each sub-component evolving relatively independently. Although skills such as planning and organized strategic behavior may manifest early, substantial progress typically occurs around ages 6–8, when children begin to regulate their behavior, set goals and anticipate outcomes, albeit with occasional impulsivity and failures in inhibitory processes. Further refinement continues into adolescence, reaching a peak around age 16 [31,32,33].

Children with 47,XXY KS show task-specific impairments in inhibitory skills despite intact planning, concept formation, problem solving, task switching and decision making under time pressure; the pattern of responses indicates inhibitory difficulties within response time tasks, suggesting that the additional X-chromosome has selective effects on cognitive phenotypes [34].

Inhibitory abnormalities may manifest as either failure to inhibit or exaggerated inhibition with a rigid focus on a subset of stimulus inputs. Weaker working memory and processing speed are common in both specific learning disorders and KS [35]. Boone et al. (2001) showed that executive function impairments in KS adults were more pervasive and pronounced compared to language weaknesses [36].

The neurobiological processes underlying the neuropsychological profile of KS remain largely undefined. Structural and functional brain differences, particularly in auditory, motor, language and social processing regions, contribute to varied neuropsychological outcomes in KS [37].

Recent research found that non-mosaic KS adolescents with impaired executive function exhibited reduced activation in brain areas crucial for executive function, including the inferior frontal gyrus, anterior insula, dorsal anterior cingulate cortex and caudate nucleus. The severity of pubertal delay was associated with the extent of these activation differences [38].

Van Rijn and Swaab (2020) linked executive function deficits to emotional dysregulation in 47,XXY KS individuals, particularly noting the associations between poor emotion regulation and diminished cognitive flexibility and attention [39]. Atypical emotion regulation strategies, including heightened emotional expression, avoiding, distraction seeking and passive coping, were observed. Inhibitory control deficit was also associated with emotional outbursts, which in turn were correlated with anxiety, depression, thought problems and hostility [37].

### 2.4. Emotional Problems

The prevalence of psychiatric disorders in KS surpasses the rates observed in the general male population, although research is limited by sample size [12,16]. Depression rates among KS men range from 19% to 69% [40,41]. Follow-up studies revealed increased psychiatric referrals and diagnoses, notably depression, among XXY individuals compared to controls [15,42]. Emotional arousal difficulties and challenges in emotional expression were noted, potentially contributing to the under-reporting of psychiatric symptoms in KS [23,43,44,45,46,47,48,49,50]. Psychiatric screenings among the KS population showed elevated rates of psychosis and depression [40,51]. Turriff et al. (2011) evidenced depressive symptoms’ prevalence of 69% among 310 XXY individuals aged 14–75 years [41]. Structural brain differences [52,53] and X-chromosome gene overexpression may influence social cognition and susceptibility to psychiatric disorders [40,54,55]. An association between hypogonadism and depression has also been suggested, although the evidence is controversial [56,57,58]. Similarly, anxiety disorders are prevalent among KS individuals, affecting behavior and function [59,60,61].

## 3. Case Report

We report the case of an 11-year-old child with prenatally diagnosed mosaic KS, confirmed postnatally as 46,XY/47,XXY mosaicism. His medical history was uneventful until the end of his first year of junior high school, when teachers observed declining academic performance, excessive daytime fatigue and distractibility, especially in recent months. Described as shy, particularly outside his household, the child experienced no psychological distress until the past year, when difficulties falling asleep, night terrors and daytime sleepiness emerged alongside his parents’ divorce and two co-occurring SARS-CoV-2 infections. Family history revealed a predisposition to psychiatric disorders, including bipolar and depressive disorders in paternal and maternal lines, respectively, with the child’s 15-year-old sister affected by obsessive compulsive and eating disorders.

Initially, the child displayed watchfulness, spatial and temporal orientation, clumsiness, embarrassment, maintaining a rigid posture and avoiding eye contact. Facial expressions showed sadness; the speech was brief and limited but responsive to prompts; and the tone was subdued. Though initially unable to express emotions openly, the child identified the beginning of middle school and parental divorce as concomitant major stressors in the fall of the previous year, leading to pronounced sadness and shame, increasing isolation both at home and school. Concerned about peer judgment, he avoided social interactions, especially with the opposite sex, feeling inadequate, especially about his body. His parents hinted at something about KS, but without further explanation.

**Figure 1:**
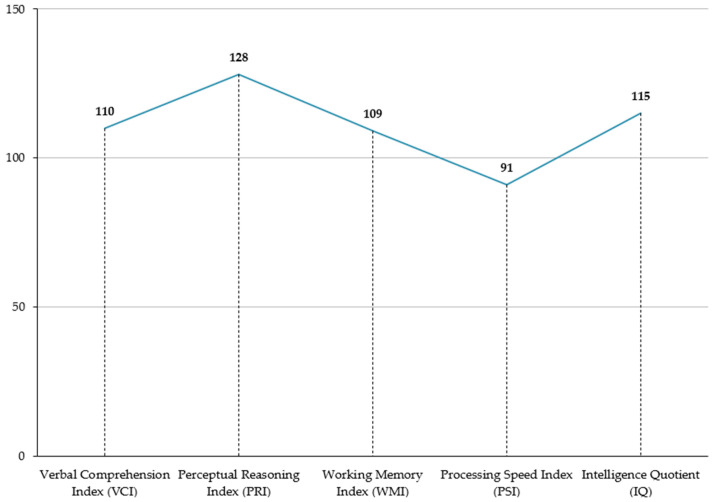
Wechsler Intelligence Scale for Children—IV edition (WISC-IV, 2003): child cognitive functioning and trend of indices. See Table 1 for the percentile ranks of test scores.

The Wechsler Intelligence Scale for Children—IV edition (WISC-IV) [62] was administered to assess the child’s cognitive abilities, revealing a FSIQ of 115 (above average). However, there were discrepancies among its primary indices: the Verbal Comprehension Index (VCI) of 110 was deemed unreliable due to performance variations in Similarities and Vocabulary subtests; the Working Memory Index (WMI) subtests showed discrepancies as well; the Perceptual Reasoning Index (PRI) and Processing Speed Index (PSI) were above average and average, respectively (Table 1) (Figure 1).

The assessment of academic skills found no specific learning disorder, albeit there was an underperformance in reading and comprehension, suggesting a potential vulnerability to difficulties in that domain. The child exhibited adequate executive abilities in the areas of planning, problem solving, strategy utilization and transfer tasks, along with maintaining attention and behavior, as demonstrated by the Tower of London test [63], wherein the child was required to mentally plan a sequence of moves to match a set of discs and execute them sequentially.

Inhibitory skills were assessed using NEPSY-II [64] inhibition subtests, involving identifying shapes or arrows based on color, with increasing difficulty. The child exhibited deteriorating performance under heightened demands, struggling to inhibit impulsive responses promptly. Verbal working memory was weak, while visual working memory appeared adequate based on NEPSY-II Memory and Learning subtests.

**Figure 2:**
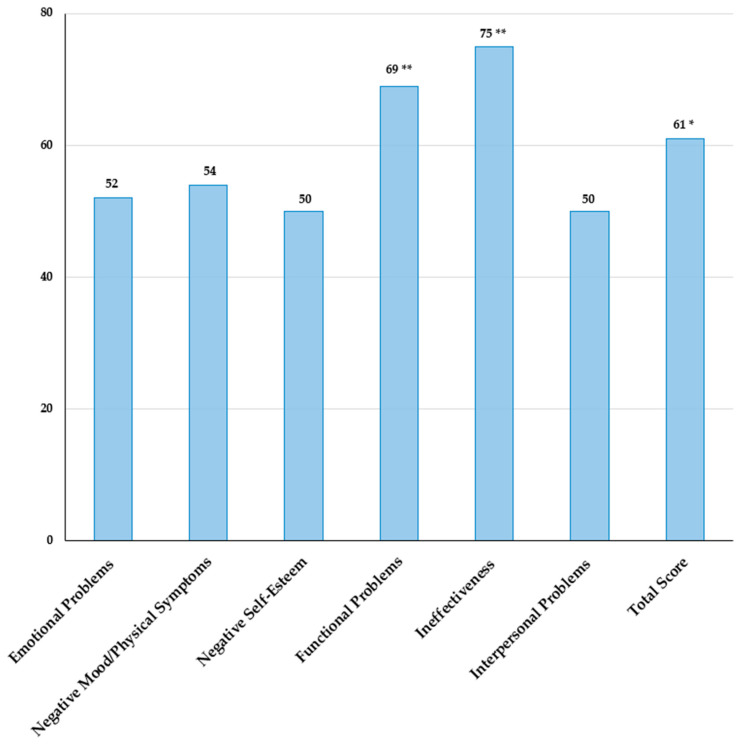
Children’s Depression Inventory (CDI) 2 self-report: identification of depressive symptomatology referred by the child and its severity. Normal: T-score ≤ 59; borderline: T-score 60–64 (*); clinical: T-score ≥ 65 (**).

Depressive symptoms were evaluated using the Children’s Depression Inventory (CDI) 2 self-report [65], indicating higher scores on the Functional Problems scale and Ineffectiveness subscale, reflecting social and academic maladjustment (Figure 2). A persistent depressive disorder, along with subthreshold generalized anxiety disorder, represented the major diagnoses through the Kiddie-Schedule for Affective Disorders and Schizophrenia-Present and Lifetime Version (K-SADS-PL) [66] semi-structured interview with the child and parents.

**Figure 3:**
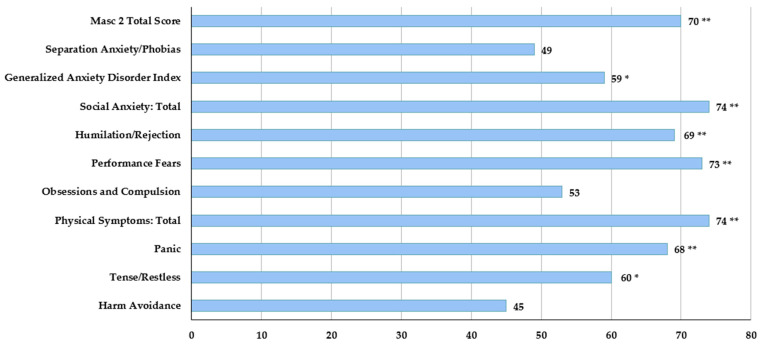
Multidimensional Anxiety Scale for Children 2nd Edition (MASC 2): an estimate of anxiety symptoms through a self-report questionnaire. Normal: T-score ≤ 54; borderline: T-score 55–64 (*); clinical: T-score ≥ 65 (**).

Anxiety symptoms were prominent, as evidenced in the elevated scores on the Multidimensional Anxiety Scale for Children 2nd Edition–Self-Report (MASC 2–SR) [67], particularly in social anxiety, humiliation/rejection, performance fears and physical symptoms (Figure 3).

The parents’ report-based Sleep Disturbance Scale for Children (SDSC) [68] showed a “borderline” total score (T = 69), with a clinically high score regarding “disorder of excessive somnolence” (T = 92).

Parent-reported questionnaires indicated clinically significant anxiety (T = 71) on the Child Behavior Checklist (CBCL) Diagnostic and Statistical Manual of Mental Disorders-Fifth Edition (DSM-5)-oriented scales [69], and cognitive issues (T = 100) and ADHD (T = 89) on the Conners’ Parent Rating Scale-revised (CPRS-R, short version) [70]. Teachers echoed similar concerns on the Teacher’s Report Form (TRF) [69] and Conners’ Teacher Rating Scales-revised (CTRS-R, short version) [71].

Persistent depressive disorder and generalized anxiety disorder were diagnosed, prompting individual psychotherapy targeting socialization, peer comparison and opposite-sex interactions, with secondary goals to prevent social withdrawal and foster identity development—crucial, given the impact of puberty on self-evaluation. Relational and communicative issues between parents hampered the child’s emotional needs; therefore, parenting support was advised to address familial conflicts and maintain attachment amid separation, and a six-month family follow-up program was initiated.

## 4. Discussion

Beyond illustrating the complex characteristics of KS and its psychological features in children, this narrative review and case report aim to emphasize some of its important aspects within pediatric endocrinology and neuropsychiatry in order to develop targeted interventions, which address the specific requirements of pediatric KS patients effectively.

In addition to the classic 47,XXY form (80–90% of KS cases), other subclasses, such as mosaicism (primarily 47,XXY/46,XY), comprise the remaining 10–20% [1,72]. The 47,XXY karyotype arises from spontaneous non-disjunction of paired X-chromosomes during germ-cell meiosis, spermatogenesis or oogenesis, while mosaicism stems from postfertilization non-disjunction [73]. The true prevalence of mosaic forms might be underestimated due to factors such as mosaicism being confined to the testes [74] or enhanced androgenization in mosaic KS individuals [73]. Mosaic KS has been proposed as a potential factor in psychiatric and neurodegenerative diseases, necessitating vigilant monitoring for the onset of multifactorial brain diseases in adulthood. Exploring the neuromarkers associated with aging in KS is also warranted [75,76,77].

The presence of an additional X-chromosome in males significantly influences brain function, leading to manifestations ranging from mild cognitive challenges to severe neuropsychiatric disorders [2]. Previous research indicates that individuals with higher grade aneuploidies present with a more severe phenotype [11], whereas 47,XXY/46,XY mosaics typically manifest fewer clinical signs and symptoms [78]: larger mean testicular volumes, milder endocrine irregularities and a lower incidence of azoospermia and gynecomastia are usually found [73,74]. With regard to neuropsychological issues, Vorsanova et al. (2022) evaluated KS mosaicisms among boys with neurodevelopmental disorders, finding rates of KS-associated karyotypes and KS mosaicisms of 1.1% and 0.6%, respectively [75]. However, the dynamic nature of KS mosaicism complicates phenotype–karyotype correlations, indicating inconsistent associations between mosaicism rates and phenotypic outcomes. As no direct associations between specific phenotypes and karyotypes were identified, the authors posited that KS mosaicism likely serves as a component within the pathogenetic cascade rather than being a primary genetic determinant of phenotypic expression [75]. Many other stressors and environmental factors, when present in subjects with KS, must be taken into consideration as potential contributors to the pathogenesis of the disorders described above. Generally, KS carries an increased risk of cognitive difficulties and susceptibility to psychopathology, impacting social, academic and familial functioning and quality of life. These alterations may be already significant in prepubertal boys when compared to peers, as recently reported [79]. Our patient’s neurocognitive profile was average, as typically described in boys with 47,XXY KS. However, there was a strength in verbal comprehension and perceptual reasoning, with superiority of PRI over VCI, alongside a pronounced weakness in working memory and processing speed. While this pattern resembles that often observed in children with specific learning disorders [35], in our case, we did not report a reading disorder, since the patient achieved low but sufficient performance levels in reading and comprehension. This outcome could potentially be attributed to the child’s struggles in managing anxiety and retrieving information from memory.

Neuroendocrine dysregulation is a hallmark feature of KS, with alterations observed in various hormonal pathways. The intricate interplay between neuroendocrine modifications and psychopathological vulnerabilities in pediatric KS is multifaceted. Testicular malfunction with decreased testosterone production is typical, leading to disrupted hypothalamic–pituitary–gonadal axis feedback mechanisms. Testosterone deficiency, a hallmark feature of KS, has been implicated in the pathogenesis of psychiatric disorders [27].

There are suggestions that early testosterone therapy might enhance psychological development in individuals with KS [80], since testosterone plays a crucial role in brain development and neurotransmitter regulation. Nonetheless, the available data lack sufficient quality to advocate for this procedure for all children with KS [1,81]. The cognitive impairment, emotional dysregulation and behavioral disturbances observed in pediatric KS patients could stem from the psychosocial consequences of learning disabilities and, later on in life, from untreated androgen deficiency. Testosterone therapy may positively impact cognitive functions, although the effects on men with normal testosterone levels are less robust (males with mosaic KS appear to be more androgenized, as mentioned above), and efficacy in clinically hypogonadal men remains to be fully investigated [82]. Generalized anxiety rates are estimated at 12–18% in KS males [14,51], with testosterone treatment potentially alleviating symptoms early in life [83]. However, negative effects and neuroticism traits were reported during testosterone treatment in KS men [84,85,86].

Additionally, abnormalities regarding growth pattern, thyroid and adrenal function, which may influence neuropsychological functioning, have been documented in individuals with KS.

Recent research described a prevalence of Hashimoto’s thyroiditis of 9% in patients with KS [87]; this is in line with that derived from previous data (7% among individuals with KS, similar to that of the general male population [88]). In the same study, significantly higher prevalence of nodular thyroid disease in 122 KS adults (31%) in comparison with matched controls (13%) was found. The increased risk of nodular thyroid disease in KS has been attributed to low levels of free thyroxine, with inappropriate thyrotropin secretion, as well as to genetic instability [87]. A retrospective longitudinal investigation, with the aim of characterizing the hypothalamic–pituitary–thyroid axis in KS patients across their lifespan, confirmed lower thyroid hormone levels in KS individuals, not accompanied by a corresponding rise in thyrotropin concentrations. This study showed, for the first time, that the reduction in free thyroxine begins during the prepubertal period and persists into adulthood [9]. Chromosomal instability deriving from the additional X-chromosome, with consequent imbalance in gene transcription levels, ultimately results in misfolding and aggregation of proteins, potentially representing one of the contributing factors to both thyroid and neuropsychological pathology [89].

Boys with KS display accelerated growth from early childhood, manifesting prior to any discernible biochemical indications of compromised testicular function during pubertal development. Consequently, it is probable that additional factors, such as disparities in gene expression attributable to the underlying chromosomal aberration, substantially contribute to the observed excessive growth phenomenon [90]. The diminished testosterone/estradiol levels conventionally associated with hypogonadism, not impeding long-bone growth through the induction of epiphyseal growth plate fusion, might not be the primary factor at play. Conversely, the augmented body height could plausibly be attributed to an overexpression of growth-associated genes, such as short stature homeobox (*SHOX*), given that individuals with 47,XXY karyotypes harbor three copies of this gene [91]. An extra copy of the *SHOX* gene and all associated regulatory elements would be expected to determine overexpression; nevertheless, duplications of the entire *SHOX* gene but only part of the regulatory region have no certain genotype–phenotype correlations, exhibiting a wide range of possible phenotypes [92]. Moreover, Tropeano et al. (2016) demonstrated that *SHOX* microduplications represent a low penetrance risk factor for autism spectrum disorders and related neurodevelopmental conditions in males and females [93]. These neuroendocrine imbalances may exert effects on brain development and function also in KS, predisposing individuals to psychiatric disturbances [27].

Despite adrenal function being described as normal in KS [94], the literature data show that some hormonal alterations, such as those involving dehydroepiandrosterone sulfate (DHEA-S), can be detected [95]. Spaziani et al. (2018) demonstrated significantly lower DHEA-S levels in KS patients than in controls, both in subjects aged 12–20 years and > 20 years; a decline in DHEA-S concentration is among the early manifestations of late-onset hypogonadism, commonly preceding the decline in testosterone values [95]. The age-related reduction in DHEA-S levels is known to be associated with various phenomena, including depression, other mood and eating disorders, as well as chronic stress states [96].

Research has demonstrated that adverse life events can disrupt neural networks and cognitive processes involved in executive functioning [97,98]. For instance, exposure to chronic stressors or traumatic events may lead to dysregulation of the hypothalamic–pituitary–adrenal axis, resulting in elevated cortisol levels and alterations in brain regions implicated in executive functions, such as the prefrontal cortex. As a consequence, individuals with KS may experience certain difficulties resulting in decreased mental flexibility, inhibition and impaired social cognition [43]. COVID-19 pandemic restrictions further exacerbated emotional distress and low confidence in social settings among KS subjects, who reported increasing feelings of isolation, fear and anxiety. Challenges in social communication are compounded by mask-wearing, hindering facial expression interpretation and worsening the feelings of social disconnect [99].

Furthermore, the discussion extends to the clinical implications of our findings, emphasizing the importance of a multidisciplinary approach in managing pediatric KS patients [7]. Collaboration between pediatric endocrinologists, neuropsychiatrists, psychologists and other allied healthcare professionals is essential for holistic care delivery, encompassing both the endocrine and neuropsychiatric aspects of KS. This integrated approach not only facilitates early identification of psychopathological symptoms but also enables targeted interventions aimed at optimizing neurodevelopmental outcomes and enhancing the overall quality of life for children with KS.

## 5. Conclusions

Individuals with KS are highly susceptible to psychiatric disorders, particularly depression, which significantly impacts their quality of life and increases the risk of suicide. Untreated psychiatric conditions in early childhood may escalate into multiple disorders, including personality disorders. Attention should also be paid to the potential presence of a specific learning disorder, given the typical imbalance between VIQ and PIQ, although, in light of new evidence as mentioned above, cognitive profiles in the pediatric–adolescent population with KS can be variable. Moreover, our findings highlight the need for heightened awareness among healthcare professionals regarding the neuroendocrine and psychopathological vulnerabilities associated with KS, particularly in pediatric populations. For both mosaic and non-mosaic KS children and adolescents, it is essential to thoroughly assess genetic predisposition and environmental influences to prevent the onset of psychiatric symptoms. Early identification and intervention in high-risk KS children can help mitigate mental health issues and reduce lifelong disability. Further research should explore the educational and psychosocial challenges faced by individuals with KS during pediatric and adolescent age and their impact on psychological well-being and overall quality of life in adulthood. Similarly, additional investigation should aim to identify personalized treatment strategies for these individuals, with a focus on their unique cognitive characteristics.

## Figures and Tables

**Table 1:** Wechsler Intelligence Scale for Children—IV edition (WISC-IV, 2003): child cognitive functioning. All indices were standardized for sex and age within a specific confidence interval (CI). Each descriptive category refers to the percentile rank in which the child’s performance was placed.

Subtests/IQ	Score	CI 95%	Percentile Rank	Descriptive Category	Is the Index/IQ/Cluster Interpretable?
**Verbal Comprehension Index (VCI)**	**110**	102–116	74.7	Average	No
*Similarities*	8		25.1		
*Vocabulary*	12		79.4		
*Comprehension*	15		95.3		
**Perceptual Reasoning Index (PRI)**	**128**	118–124	97.0	Above average	Yes
*Block Design*	15		95.3		
*Picture Concept*	14		90.8		
*Metrics Reasoning*	14		90.8		
**Working Memory Index (WMI)**	**109**	99–117	72.7	Average	No
*Digit Span*	15		95.3		
*Letter–Number Sequencing*	8		25.1		
**Processing Speed Index (PSI)**	**91**	82–102	28.0	Average	Yes
*Coding*	8		25.1		
*Symbol Research*	9		37.1		
**Intelligence Quotient (IQ)**	**115**	108–120	83.3	Above average	Yes
**General Ability Index (GAI)**	**120**	114–125	92.0	Above average	Yes
**Cognitive Proficiency Index (CPI)**	**100**	93–107	54.0	Average	Yes

## Data Availability

Data sharing is not applicable.
